# Burrowing activity of coconut rhinoceros beetle on Guam cycads

**DOI:** 10.1080/19420889.2020.1774310

**Published:** 2020-06-02

**Authors:** Thomas E. Marler, Frankie C. Matanane, L. Irene Terry

**Affiliations:** a Western Pacific Tropical Research Center, University of Guam, Mangilao, GU, USA; b School of Biological Sciences, University of Utah, Salt Lake City, UT, USA

**Keywords:** Adaptive radiation, CRB, *Cycas micronesica*, ecological fitting hypothesis, host shift, *Oryctes rhinoceros*, starch

## Abstract

Guam’s established population of non-native coconut rhinoceros beetle (CRB, *Oryctes rhinoceros* L.) began creating burrows in stem apices of several cycad species in a managed garden. We conducted an island-wide survey to determine the spatial patterns of CRB burrowing of stems of *in situ Cycas micronesica*. We also measured starch of healthy and unhealthy coconut leaf tissue and compared this with starch of cycad stem tissue. The starch concentration of the central unexpanded leaf in healthy Philippine coconut trees was ≈90 mg·g^−1^, and that of unhealthy Guam coconut trees was ≈40 mg·g^−1^. The starch concentration of the tissue within the CRB burrow locations on *C. micronesica* trees was 145 mg·g^−1^. Burrowing of *C. micronesica* was restricted to female CRB adults and was found throughout the full latitudinal gradient of Guam. Our findings indicate Guam’s unhealthy coconut trees are no longer nutrient-dense, and the female CRB population may have exhibited a phylogenetically distant host shift to the abundant *C. micronesica* plants for a starch-rich diet within the concepts of the ‘ecological fitting’ hypothesis. We add proximity to coconut tree habitats as a new threat to Guam’s endangered *C. micronesica* population.

## 1. Introduction

Coconut rhinoceros beetle (CRB) is a devastating pest of the economically important coconut (*Cocos nucifera* L.). The female adult oviposits in and larvae feed on dead and decaying organic materials [1–6]. The larval stage is 11–15 weeks in length, and the pupal stage is about 6 weeks in length [2]. The adult beetles emerge from the dead and decaying tissues to begin the life stage that directly consumes liquids that are expressed by macerating metabolically active soft tissues in growing plant organs [1–9]. The common name of this herbivore is misleading, in that the reported host range for the adult stage is extensive [1–9]. Adult females live up to 9 months [6].

The invasion of Guam by CRB was first documented in 2007 [10–13]. A 2002 survey of Guam’s forests indicated that coconut was the second most abundant tree species on Guam [14]. The ubiquitous coconut tree population and lack of biological control have led to a well-established CRB population throughout Guam. The same 2002 survey indicated *Cycas micronesica* K.D. Hill was the most abundant tree in Guam’s forests [14]. The indigenous range of this arborescent cycad includes the western Pacific islands from the Mariana Islands to Palau [15].

A substantial literature has accumulated on *C. micronesica* due to the invasions of several non-native insect herbivores since 2003. These included the armored scale *Aulacaspis yasumatsui* Takagi [16,17], the *Cycas*-specific *Chilades pandava* Horsfield butterfly [18], the leaf miner *Erechthias* Meyrick sp [16], and the termite *Schedorhinotermes longirostris* Brauer [19]. Sustained damage following the invasions of these non-native herbivores led to irruptions of the native stem borer *Dihammus marianarum* Aurivillius [16]. The documented and projected plant mortality led to the assignment of Endangered status on the International Union for Conservation of Nature Red List [15] and Threatened status on the United States Endangered Species Act (ESA) [20].

Active management of ESA-listed tree species should include frequent surveys by species experts to determine the status of known threats and document any nascent developments of previously unknown threats. This is especially true for *C. micronesica* due to the diversity of biological threats, and observational studies conducted by species experts should be included in all conservation projects to improve conservation knowledge as the populations continue to decline in range and density [21,22].

The University of Guam curates a collection of cycad species among several Guam locations, all of which contain mature coconut trees. We observed CRB burrowing of a *Macrozamia moorei* F. Muell. plant, then burrowing in several other species thereafter. These observations initiated several actions to more fully understand what appeared to be an unexpected host shift by CRB. The adult CRB feeds on starch-rich tissues [23], and cycad stems are documented sources of starch [24–27]. Our objectives were to determine the geographic range of CRB herbivory of *C. micronesica* throughout Guam and determine starch concentrations of healthy and unhealthy coconut tree spear leaf tissue to determine if CRB behavior may be correlated with compromised starch content of Guam’s coconut trees.

## 2. Materials and methods

### 2.1. Starch analysis

**Figure 1.**
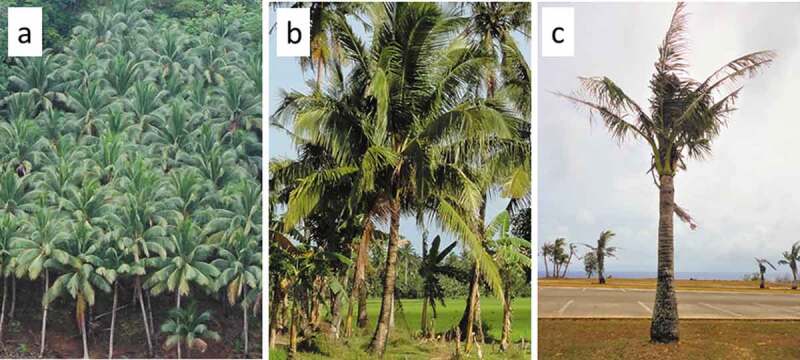
*Cocos nucifera* trees in three production and landscape settings. (a) Highly productive Philippine copra agroforest. (b) Healthy unmanaged tree in the Philippine farm setting. (c) Unhealthy tree in Guam landscape 11 y after the invasion of *Oryctes rhinoceros.*

We set out to determine if the tissue from Guam’s unhealthy coconut trees were deficient in nutritional value compared with healthy coconut trees. We focused on starch concentration for this endeavor. In each of three locations, six coconut trees were used to obtain unexpanded leaf tissue from the center of the stem apex. (1) A managed commercial copra farm in Libertad, Philippines was used to collect samples on 17 Oct. 2018 (Figure 1). (2) Several farms in Angeles City, Philippines were used to collect tissue samples on 29 Oct. 2018. These healthy coconut trees were maintained for fresh fruit production. (3) Landscape coconut trees in Mangilao, Guam were used to collect tissue samples on 5 Nov. 2018. The Guam trees were representative of unhealthy trees on the island after 11 y of damage by CRB. One core per tree was extracted at the microsite and orientation to mimic the typical burrows in the petiole bases that evince CRB herbivory. A 1.9-cm hole saw was used with a portable drill, and each core was drilled to a depth to extract the central unexpanded leaf tissue. This is the tissue that CRB adults macerate to express the liquids that are consumed. The samples were immediately dried at 75°C for 24 h.

The starch concentration of *C. micronesica* stem tissue has been determined in various strata below the leaf crown [25,26]. However, the starch concentration of the tissue immediately beneath the cataphyll complex at the apex of the stem has not been determined to date. Therefore, we used the hole saw to extract tissue to a depth of 4 cm from the apex of six *C. micronesica* plants in an *ex situ* cycad collection in Angeles City, Philippines. The plants were part of our biodiverse cycad collection, were 7-y old, and were sourced from Yap. These healthy plants were used because no healthy *C. micronesica* trees are available on Guam due to the damage by the previously described non-native insects. The samples were dried for 24 h at 75°C. The resulting hole was filled with expandable insulation foam, and then the entire area was sealed with a petroleum-based pruning sealant. The trees recovered with no subsequent symptoms.

All samples were milled to pass through a 1-mm screen then starch was quantified in accordance with Marler and Dongol [28] as glucose equivalents after hydrolyzation by amyloglucosidase [29]. The coconut starch data were subjected to one-way analysis of variance with location as the factor (Proc GLM, SAS Institute, Cary, NC, USA). Means separation among the locations was conducted with Tukey’s HSD test.

### 2.2. Cycad burrowing

When the number of cycad species in the university cycad garden exhibiting signs of CRB burrowing began to increase, then the native *C. micronesica* plants in the garden were added to that list, we conducted an island-wide survey to determine if CRB damage could be found among *in situ C. micronesica* localities. We focused on 14 localities that we have studied since 2005 for various conservation projects, as we curate a substantial database from these localities. For each locality, we searched for signs of CRB herbivory in January and February 2020. We did not determine the extent of damage in each locality, as our objective was to determine the spatial patterns of documented CRB herbivory throughout the island. To create a list of localities with evidence of CRB damage, we searched each locality until 200 trees were observed or until CRB burrows were found.

When a burrow was found, we extracted the frass and animal (when present) then measured the depth of the burrow. We recorded the sex and orientation of each CRB individual within the burrows.

## 3. Results

### 3.1. Starch

**Figure 2.**
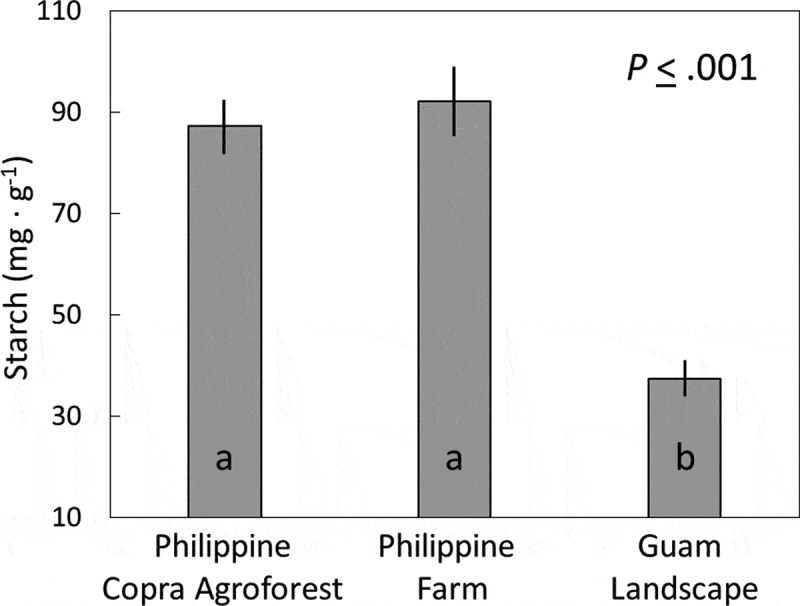
Starch concentration of the unexpanded spear leaf extracted from the apex of *Cocos nucifera* trees in three locations. Mean ± standard error, n = 6. Columns with the same letter are not different according to Tukey’s HSD test (*P* <.05).

The starch concentration of the central unexpanded leaf from healthy Philippine coconut trees did not differ between the commercial copra agroforest locality and the farm locality and was about 90 mg·g^−1^ (Figure 2). The starch concentration of the unhealthy Guam trees was less than half of that for the healthy trees. The starch concentration in the healthy *C. micronesica* stem tissue subtending the cataphylls was 145 ± 9 mg·g^−1^.

### 3.2. Cycad burrowing

Following the initial observation that adult CRB burrowed *M. moorei* stems, four more cycad species at the University of Guam were observed with CRB herbivory. In the chronological order of observation, these were *Microcycas calocoma* (Miq.) A.DC., *Cycas zambalensis* Madulid & Agoo, *Cycas micronesica*, and *Dioon edule* Lindl.

**Figure 3.**
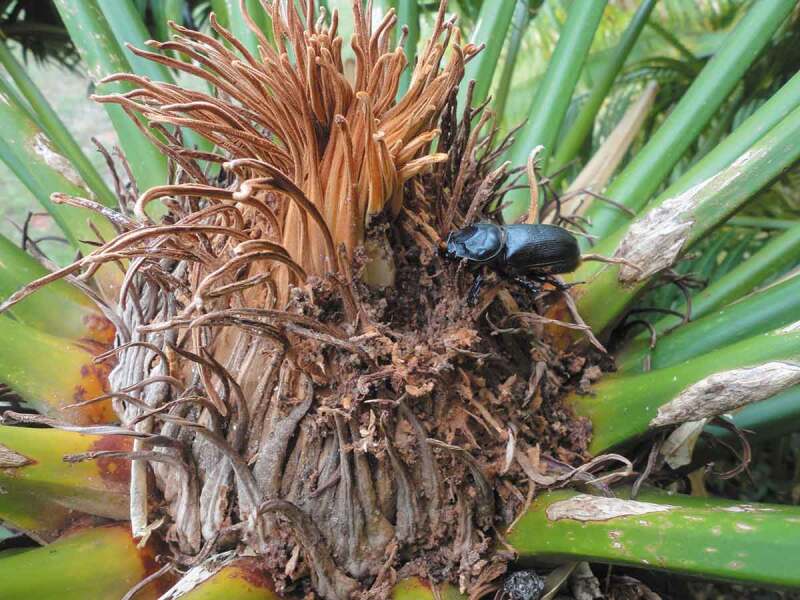
The appearance of *Oryctes rhinoceros* damage to *Cycas micronesica* cataphyll complex with the frass and female adult that were extracted from the burrow. Burrow was 7.5 cm deep.

In the four latter cases, the adult female CRB entered the cycad stem by boring a hole through the apical cataphyll complex. The opening for each burrow was unlike that for other CRB host species, in that the entry point was apical to the host tree’s leaf petioles (Figure 3).

**Figure 4.**
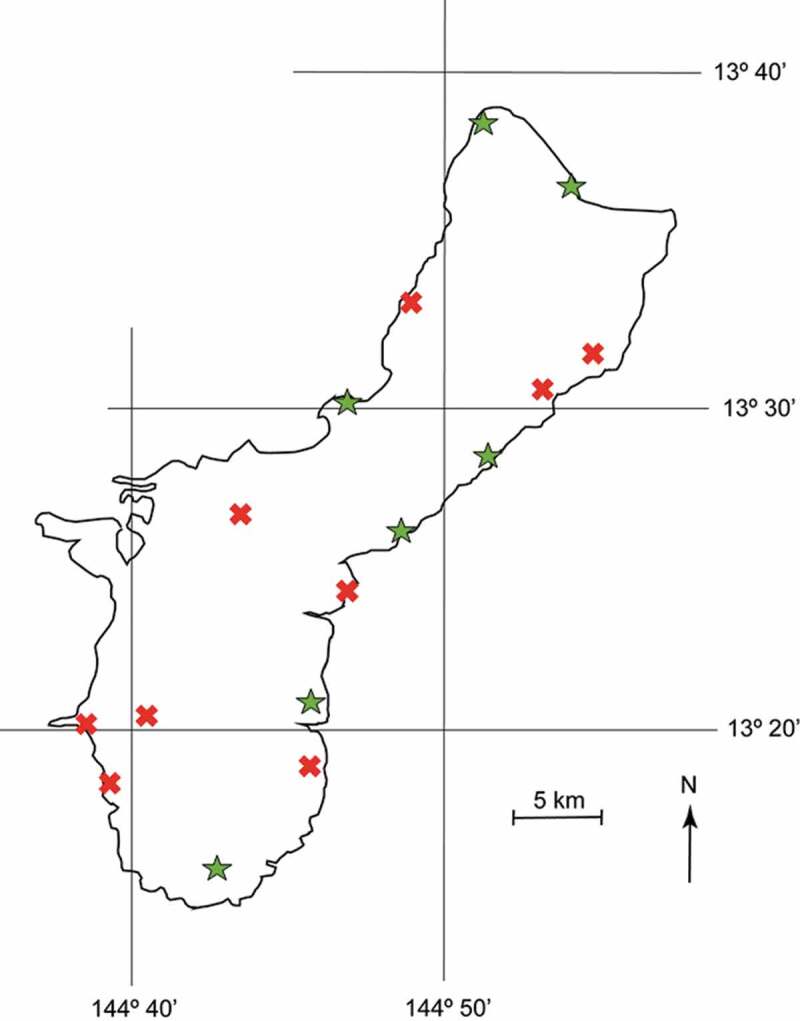
Locations on the island of Guam where *Oryctes rhinoceros* burrowing of *in situ Cycas micronesica* trees were confirmed in January–February 2020. Green symbols were sites with verified CRB damage, red symbols were sites with no visible CRB damage among 200 observed trees.

We observed CRB burrowing in the cataphyll complex of *C. micronesica* trees in five of our 14 permanent study sites (Figure 4, the 14 southernmost sites). All five sites were nearby populations of damaged coconut trees. These sites did not include localities inside federal lands, as we have not obtained data from our previous long-term plots within a federal property since 2015 due to the imposition of new unacceptable prerequisites for accessing the sites for conservation research purposes. However, we were able to visit three *in situ* conservation plots that we constructed within the federal property during court-approved visits in January 2020, and we observed CRB damage at two of these plots (Figure 4, the two north sites). Therefore, the current status of CRB burrowing of *C. micronesica* trees extends the full latitudinal gradient of the island.

All of the cycad burrows exhibited orthotropic orientation and were full of frass. Additionally, considerable amounts of frass had been expelled by the CRB behavior, and this collected on the cataphyll surfaces adjacent to the hole (Figure 3). The feeding adult CRB individual was in the burrow for 85% of the observed trees, and in every case the adult was a female. The depth of the burrows ranged from 6 to 8 cm, and no eggs, larvae, or secondary tissue necrosis were observed in the fresh burrows.

## 4. Discussion

### 4.1. The plants

The two most abundant trees in a 2002 survey of Guam’s forests were *C. micronesica* and coconut [14]. These two ecologically and culturally important trees experienced no serious phytophagous insect threats prior to the invasions that began when *A. yasumatsui* was first documented on Guam in 2003 [17]. In that same year, the leaf miner *Erechthias* sp. was also first identified on Guam [16]. The *Cycas*-specific butterfly *C. pandava* was first observed in 2005 [18] and CRB was documented in 2007 [10–13].

Prior to these invasions, Guam’s *C. micronesica* plants were the host for a native stem borer *D. marianarum*, and signs of stem herbivory could be observed on rare occasions prior to 2003 [16]. The temporal patterns of damage to Guam’s *C. micronesica* trees were not similar for each of these herbivores during the years after the *A. yasumatsui* invasion [30]. For coconut, several non-native scale and beetle herbivores caused mild to moderate damage, but no lethal arthropod threat preexisted the CRB invasion. Since 2003, no overlap in the threats or correlation of conservation needs has occurred for these two important tree species until now. The behavior change by CRB has thrusted Guam into a new era, as we propose the proximity of coconut tree habitats to *in situ C. micronesica* trees as an unprecedented threat to Guam’s cycad population.

### 4.2. Why only females?

The host range for adult CRB is extensive [1–9], so the discovery of a previously unknown host species is not surprising. But to our knowledge this is the first report that indicates the burrowing activity of adult food material is restricted to one CRB sex. One explanation for this observation is that the burrowing is not for herbivory but is instead for the preparation of oviposition sites. Reported CRB larvae foods have included a plethora of dead and decomposing materials including standing dead trees, mulch, compost, suspended arborescent litter, untreated lumber, and bovine scat [1–6]. We note that there has never been a report of larvae feeding on metabolically active growing plant tissues within living plant organs. Reported CRB adult foods have included a long list of plant species [1–9], and we note that in every report the adults feed on metabolically active growing plant tissues within living plant organs. The interpretation of burrowing in growing cycad stem apices is for oviposition sites is in conflict with the CRB herbivory literature, but the interpretation that this burrowing is for herbivory is in conformity with the CRB herbivory literature. The CRB larvae are voracious feeders, and our repetitive observations of the identified burrows have never documented signs of larvae feeding around the burrows. A second explanation for the female-only burrowing activity is that the energy needs for female adults exceed those of male adults, and the host shift is in response to these energy needs. We discuss this as the most plausible explanation in the following subsection.

### 4.3. Why now?

Our selection of starch to define the nutritional quality of various tissues was based on reports indicating amylase is one of the dominant digestive enzymes for adult CRB [23]. Moreover, several published reports were available concerning *C. micronesica* stem starch, and these reports were accessible to aid in interpretation. For example, starch concentrations of 160–200 mg·g^−1^ have been reported within stems of healthy *C. micronesica* or *C. revoluta* plants [25–27]. Moreover, starch concentrations of 120 mg·g^−1^ have been reported for unhealthy *C. micronesica* plants following 7 years of damage by non-native *A. yasumatsui, C. pandava*, and *Erechthias* sp [27]. Herein we have reported starch concentrations of 145 mg·g^−1^ for stem tissue subtending the apical cataphylls of healthy *C. micronesica* plants in an *ex situ* garden. All of these values for *Cycas* stem starch greatly exceed those of the healthy coconut tissue (Figure 2).

Observational studies on islands like Guam may emerge as interesting case studies due to their insular settings [31,32]. The sessile coconut trees on Guam have been unable to escape the relentless damage by CRB since 2007. Moreover, the attempts at introducing CRB biological control agents have been minimally effective to date [12]. We propose that these traits of Guam’s coconut and CRB populations have slowly created a circumstance where the nutritional density of the typical CRB diet has declined below a threshold such that the CRB have shifted to exploit the opportunistic availability of the starch-rich cycad stems. The fact that 100% of the CRB individuals we extracted from the *C. micronesica* tissue were female may indicate that their energy needs for the production of healthy eggs and construction of the oviposition sites exceed the energy needs of the males.

Proximity to *C. micronesica* habitats may be considered a new treat for Guam’s remaining coconut trees if our interpretations are accurate. Indeed, the starch-rich diet of the gravid female CRB adults feeding on *C. micronesica* tissue may increase fecundity and magnify the CRB population in areas of abundant *C. micronesica* plants.

### 4.4. Host shifts

The reported host range for adult CRB is lengthy and includes *Ananas comosus* (L.) Merr., *Colocasia* Schott sp., *Cyathea* J.E. Smith sp., *Musa* L. sp., *Pandanus* Parkinson sp., *Saccharum* L. sp., and numerous palm species [1–9]. To our knowledge, this is the first documentation that CRB has exhibited a putative shift in host range to add a cycad species. The burrowing within cycad tissues that we documented has included four genera and two families to date, so the dietary needs that are putatively being met by the cycad tissues appear to be exhibited at the Order level, not at the family, genus, or species level. We do not know if this newly documented burrowing damage will become a chronic threat to the host plant or if the widespread damage that we documented in January and February 2020 will become an ephemeral occurrence that will subside.

Host plant shifts and range expansions occur by herbivorous insects, and the concept is covered in a large body of literature involving both theoretical and experimental work. Classical coevolutionary perspective with host shifts occurring among phylogenetically related hosts focuses on co-adaptions and reciprocal evolution by both organisms [33]. Because phylogenetically related hosts might meet the requirements (e.g. nutrients, olfactory signals, plant defenses) of the herbivore more than distantly related plants, the cause of the host shift may superficially appear to fit with coevolutionary or evolutionary processes. Therefore, numerous studies have questioned the validity of such restrictive causes [see 34 for review]. One alternative to coevolution or evolutionary processes presumes that novel host shifts by parasites or herbivores could result from ‘ecological fitting’ [34,35], where shifts are due to compatible resources and signals found in the new host, and not strictly by coevolution or evolution processes. This relieves the restriction of herbivores shifting only to closely related hosts, and the shift may occur rapidly rather than in gradual stages. In addition to ‘ecological fitting’ theory in herbivore shifts to a novel host, there has been an emphasis on testing agreement with the ‘preference-performance’ hypothesis [36], that is, whether the adult female selects traits she prefers (for feeding and oviposition) that are correlated with larval development success. Host selection based on larval brood site performance is not relevant for CRB because adult females oviposit in and larvae develop on dead or decaying organic materials [1–6]. In the case of CRB, the selection of a living host strictly concerns adult feeding preference.

This CRB host selection would involve plant traits such as familiar olfactory signals, initial taste that is palatable, accessible nutrients, and suitable nutrient balance. The selection would also require no untoward traits such as repellents or toxicants that would hamper any of the attractant processes. This aspect of host shifts is why we were surprised to observe ongoing CRB herbivory of several cycad species, as this is the first time that its host taxa are known to contain numerous animal toxins. Indeed, the toxicity of cycad tissues to herbivores is a well-known trait of the Order [24]. Specialist cycad herbivores use specialized traits to sequester or detoxify the cycad tissue [37], a process that may include a common core of gut microbiota [38]. In CRB adults, the nutrients/resources could be important for maintenance, such as for flight and movement, for mating (developing pheromones, for example), or for egg development and oviposition processes. In CRB, adult females feed on starch-rich nutrients associated with soft tissues at the base of developing leaves of primarily palms. The known hosts are mostly phylogenetically related because of the predominance of palms, and this could be construed to match ‘diffuse coevolution’ among phylogenetically related palms. However, the beetle’s selection of non-palms including cycad crown tissues appears to agree with the ‘ecological fitting’ hypothesis due to the common resources, as an adaptive radiation among these diverse and unrelated plant hosts.

### 4.5. Coconut and cycad herbivory

The burrowing behavior of adult CRB is highly contrasting for coconut and cycad trees. The evidence of frass from the burrows is easily observed on the surface of the cycad cataphylls but is difficult to observe in attacked coconut trees. The burrows exhibit plagiotropic orientation for coconut trees, but orthotropic orientation for cycad trees. The actual tissue that is burrowed is leaf tissue for coconut trees but is apical stem tissue for cycad trees. The energy and time required for a CRB adult to bore into the region where soft unexpanded coconut leaves positioned are substantial. The energy and time required for a CRB adult to bore through the soft cycad cataphylls into the soft subtending parenchyma tissue are minimal. Indeed, the burrows in Guam’s *C. micronesica* trees exhibited a maximum depth of 8 cm, but the burrows in coconut trees may be up to 50 cm in length [2].

### 4.6. Non-native cycad pests and plant phenology

The four non-native herbivorous insects that have invaded Guam and caused the ESA listing of *C. micronesica* each interplay with plant phenology in a unique manner. The greatest chronic threat to *C. micronesica* is *A. yasumatsui*, which can infest the surface of any soft tissue. The crawlers of this lethal pest can navigate to any exposed organ and begin feeding. The threat of infestation of cycad leaves and female reproductive structures that are years in age is no less than those same structures that are days in age. The larvae of the specialist butterfly *C. pandava* can inflict devastating damage by tissue consumption; however, the only *C. micronesica* tissue that is palatable is young, expanding tissue. Therefore, the gravid adult butterfly needs to find a young leaf and oviposit early enough in that leaf’s expansion such that the larvae can reach pupation before the leaf tissue becomes mature. This requirement for successful herbivory causes increases and decreases in the plant population damage based on the timing of behavior of the two organisms. The *Erechthias* leaf miner oviposits exclusively on mature *C. micronesica* leaflets of old leaves, and the larvae tunnel within the mesophyll tissue in a manner that does not kill the leaflet. The fact that this leaf miner does not damage a leaf until late in the life of the leaf renders the damage from this pest as non-lethal.

**Figure 5.**
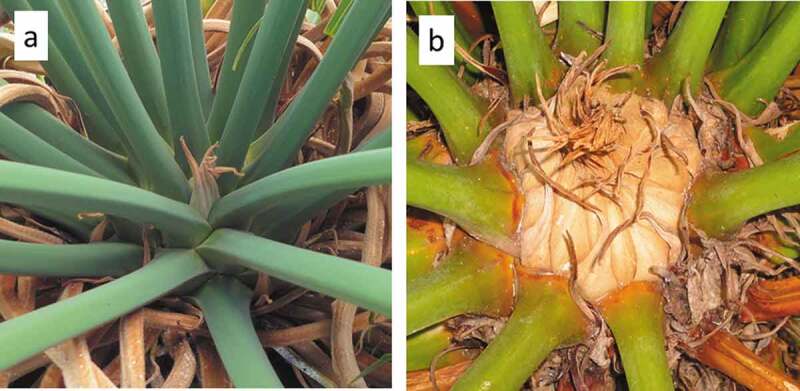
The appearance of the *Cycas micronesica* stem apex. (a) Leaf petioles are in close proximity immediately after a vegetative flush, and the apical cataphylls are diminutive. (b) Over several months the leaf petioles are pushed apart by the developing cataphyll complex, and immediately prior to a new organ flush the apical cataphylls are robust.

The CRB burrowing behavior we have observed is restricted to the phenological stage immediately prior to an impending primary growth pulse. This may be a physical exclusion phenomenon, in that the access to large spaces on the cataphyll complex is difficult immediately after a leaf flush (Figure 5(a)). Over several months, the cataphyll complex becomes mitotic and robust as a means of preparing for the subsequent expansion of new leaves or strobili. All of the CRB burrows we observed were on trees with the robust, developed cataphylls providing ample space between the leaf petioles for the animal to burrow (Figure 5(b)). These nuances indicate that management approaches for the CRB herbivory may exploit the herbivore’s observed requirement of appropriate cycad plant phenological stage. Scouting and interventions may focus on *C. micronesica* trees containing large cataphyll structures on trees that have experienced many months of development since the antecedent primary growth pulse. Although growth pulses among Guam’s *C. micronesica* population may occur during any month of the year, more vegetative growth pulses occur in August than any other month, and more reproductive growth pulses occur in April than any other month [39]. This knowledge indicates a high percentage of the tree population may exhibit the appropriate stem apex phenotype for CRB burrowing during March and July.

### 4.7. Cycad conservation efforts

This case study illuminates the value of *ex situ* germplasm collections for adaptive management improvements in tree conservation. The need to understand the starch relations of *C. micronesica* tissues subtending the apical cataphylls could not be predicted prior to 2018 when the CRB burrowing activity was first observed. Our healthy off-site *C. micronesica* collection in the Philippines provided a source of tissue to answer the urgent questions surrounding why CRB may exhibit the unexpected host shift to cycad species.

The first federal contract involving field *C. micronesica* research and conservation occurred in 2001 (to T.E.M.). Numerous contracts were awarded thereafter, and the practitioners who managed these funded projects from 2001 to 2015 were actively involved in contributing to the cycad primary literature. Every project resulted in advances of relevant conservation knowledge and generation of informative refereed journal publications. This approach to funding *C. micronesica* conservation efforts emerged as an example of how improvements in adaptive management to inform future conservation decisions are achievable whenever funding agencies look to international experts to meet their needs [21].

A shift in funding approaches occurred in 2015 when *C. micronesica* was listed under the U.S. Endangered Species Act [20]. The conservation teams that have been permitted by the U.S. Fish & Wildlife Service and funded by the U.S. Department of the Navy since 2015 have not contributed to the cycad primary literature in any capacity. This new approach for funding *C. micronesica* conservation ensures that the co-production of new knowledge that can withstand the filter of the peer-reviewed primary literature will be absent from ongoing conservation projects. The approach increases the likelihood of conservation mistakes because of the absence of requisite biology and ecology knowledge by the practitioners and is in sharp contrast to international recommendations for how to approach plant conservation [40–42].

The extent of CRB herbivory that we observed throughout the island during our rapid 2020 survey was unexpected because copious federal funds have sustained chronic *C. micronesica* conservation activities since 2015. Despite the sustained expenditures of conservation funds, the widespread CRB burrowing damage throughout Guam’s *in situ* populations has not been reported to date. In contrast, *A. yasumatsui* was identified in one western Guam locality in 2003, *Erechthias* sp. was identified in one southern Guam locality in 2003, *C. pandava* was noticed in one northern Guam locality in 2005, and *A. yasumatsui* was noticed in one northern Rota locality in 2007. Island-wide surveys by international cycad experts immediately after identification of these outbreak sites revealed no secondary infestation sites, so the conservation teams were able to follow the patterns of damage to each invasive pest from the initial locality to the remainder of the localities. A plan to follow the patterns of CRB damage to the widespread *C. micronesica* localities is no longer possible because the CRB host shift is well established and has been unreported by the funding agencies or the funded practitioners.

### 4.8. Immediate conservation needs

An immediate survey is needed to determine the extent and spread of CRB damage to the native *C. micronesica* populations throughout Guam and Rota and confirm the exclusive burrowing entry point is the cataphyll complex. *Bona fide* experienced cycad biologists are needed to fulfill this endeavor with accuracy. The symptoms of the cataphyll complex following the CRB burrowing behaviors are not easily identified if one does not have full knowledge of cycad morphology and phenology, which may be the reason this added biological threat to the native cycad population has proceeded without notice. We have observed that the burrows close visually within 2–3 d after the animal is removed, and we assume this same plant behavior occurs after an animal vacates a burrow voluntarily. The visual closure is caused by a collapse of the cataphylls around the hole such that the radial symmetry that defines a healthy cycad stem apex is compromised. Knowledgeable cycad biologists with germane expertise would be able to readily identify these nuances during *in situ C. micronesica* surveys. Conservationists in all other geographic regions where *in situ* cycad populations coexist with CRB-damaged coconut trees should remain vigilant in looking for a similar host shift, especially if the coconut population exhibits compromised health.

Long-term plant behavior following CRB damage should be monitored by experienced cycad biologists. The results of these observations will determine if the nascent CRB damage is a new acute threat for the attacked trees or just one more minor pest that has been added to the list of biological threats that are slowly causing the demise of the *C. micronesica* plant population. We predict the CRB damage will include subsequent loss of the stem apex due to secondary infections. Indeed, when the periderm and bark of *Cycas* stems are broken to expose the stem’s parenchyma, subsequent necrosis can advance into the stem tissues until a localized adventitious phellem is formed to seal off the necrosis [43]. We predict the orientation of the bored holes may magnify this necrosis of the exposed parenchyma tissue, as rainfall will accumulate in the open orthotropic burrows and promote greater tissue damage. Secondary pathological infection is one of the means by which CRB herbivory can kill the apex of coconut trees [2]. Coconut and *C. micronesica* trees lack axillary buds, so the development of lateral stems after the loss of the apex is not possible, and this leads to tree mortality for the damaged coconut tree. However, *C. micronesica* trees possess the capacity to develop adventitious buds and can recover from the loss of the stem apex. Therefore, if CRB herbivory does lead to widespread loss of stem apices due to secondary infection of pathogens in the open wounds, the damage to the cycad trees may not be lethal. A knowledgeable cycad biologist is needed to conduct the observations concerning these tree responses to develop conservation mitigation actions.

The services of a biochemist or molecular biologist could provide an indirect approach to quantifying cycad herbivory among the adult female CRB population on Guam. The phylogenetic separation of coconut and *C. micronesica* is extreme, and identification of chemical and molecular markers that would evince a diet of either species could be used to determine the percentage of trapped female CRB that reveal a history of cycad consumption.

Unanswered questions abound concerning how CRB individuals respond following cycad burrowing. Since CRB is not a cycad specialist, how will the cycad toxins influence egg development and the lifespan and fitness of the CRB offspring? Do richness and diversity of CRB gut microbiota differ for individuals exclusively feeding on coconut versus individuals feeding on cycad tissues? If CRB individuals feeding on cycad tissues exhibit the common core gut microbiota of cycad specialists [38], have these gut microbes been recently acquired?
